# Improved Predictions of the Geographic Distribution of Invasive Plants Using Climatic Niche Models

**DOI:** 10.1371/journal.pone.0156029

**Published:** 2016-05-19

**Authors:** Jorge E. Ramírez-Albores, Ramiro O. Bustamante, Ernesto I. Badano

**Affiliations:** 1 División de Ciencias Ambientales, Instituto Potosino de Investigación Científica y Tecnológica, A.C., San Luis Potosí, México; 2 Departamento de Ciencias Ecológicas, Facultad de Ciencias, Universidad de Chile, Santiago, Chile; 3 Instituto de Ecología y Biodiversidad, Facultad de Ciencias, Universidad de Chile, Santiago, Chile; University of Minnesota, UNITED STATES

## Abstract

Climatic niche models for invasive plants are usually constructed with occurrence records taken from literature and collections. Because these data neither discriminate among life-cycle stages of plants (adult or juvenile) nor the origin of individuals (naturally established or man-planted), the resulting models may mispredict the distribution ranges of these species. We propose that more accurate predictions could be obtained by modelling climatic niches with data of naturally established individuals, particularly with occurrence records of juvenile plants because this would restrict the predictions of models to those sites where climatic conditions allow the recruitment of the species. To test this proposal, we focused on the Peruvian peppertree (*Schinus molle*), a South American species that has largely invaded Mexico. Three climatic niche models were constructed for this species using high-resolution dataset gathered in the field. The first model included all occurrence records, irrespective of the life-cycle stage or origin of peppertrees (generalized niche model). The second model only included occurrence records of naturally established mature individuals (adult niche model), while the third model was constructed with occurrence records of naturally established juvenile plants (regeneration niche model). When models were compared, the generalized climatic niche model predicted the presence of peppertrees in sites located farther beyond the climatic thresholds that naturally established individuals can tolerate, suggesting that human activities influence the distribution of this invasive species. The adult and regeneration climatic niche models concurred in their predictions about the distribution of peppertrees, suggesting that naturally established adult trees only occur in sites where climatic conditions allow the recruitment of juvenile stages. These results support the proposal that climatic niches of invasive plants should be modelled with data of naturally established individuals because this improves the accuracy of predictions about their distribution ranges.

## Introduction

Niche requirements of plants can vary along their life cycles because the morphological and functional traits that determine the fitness of individuals may change with plant development [1]. It was proposed that the *regeneration niche* embraces all environmental conditions that the early stages of plants require for their establishment and survival, while the adult niche includes the environmental conditions required for the survival and reproduction of mature individuals [1–4]. This conceptual framework is important to understand the spread of invasive plants because their propagation into new geographic areas mainly depends on finding suitable environmental conditions for the establishment of offspring and survival of adults [5–8].

Species distribution models that correlate occurrence records with climatic variables have become a popular method for predicting the distribution of invasive plants [9–15]. These *climatic niche models* estimate the climate thresholds within which a given species can survive and their projection on the geographical space indicates the sites where the species could be found if neither dispersal limitation nor biotic interactions are assumed [6,7,12,16,17]. Climatic niche models are then important tools in the prevention and control of plant biological invasions [14,18,19], but their application requires accurate predictions about the spatial extent that these invasions can reach. This would allow to concentrate the economic efforts addressed to manage invasive plants in those sites that are more susceptible to be colonized.

Climatic niche models are usually constructed with occurrence records of mature plants because these data are largely available in literature and collections [4,10,13,14,17]. However, because juvenile stages of plants usually have narrower tolerance thresholds than mature individuals when they are subjected to similar environmental conditions, the regeneration niche of plants may be smaller than the adult niche [1,3]. Thus, although climatic niche models based on occurrence data of mature individuals are useful to predict what sites can be occupied by invasive species (*i*.*e*. the adult niche), they may overpredict their expected distribution ranges. This is particularly valid if the survival of juvenile stages of these plants is regulated by more restrictive climatic conditions than those regulating the survival of adults (*i*.*e*. regeneration niche). Therefore, it can be proposed that the potential distribution ranges of invasive plants can be more accurately predicted through their regeneration climatic niches, which should be modelled with occurrence records of juvenile stages only.

Another important concern about the accuracy of these climatic niche models is related to the origin of the occurrence records. Most data gathered from literature and collections referring to the distribution of plants usually do not discriminate between occurrences of naturally established individuals and man-planted individuals. Nevertheless, this discrimination of the data is important for modelling climatic niches of invasive species because, if people subsidize their establishment by either economic or cultural reasons, they may be occurring in sites where climate does not necessarily favour their survival [20]. On this issue, Sax *et al*. [21] have recently proposed that including human-subsidized occurrences into climate niche modelling procedures can bias the estimation of the distribution ranges of invasive species. Thus, to improve the accuracy of the predictions obtained from these models, climatic niches should be estimated with data of naturally established individuals, while human-subsidized records should be avoided. However, because climatic niches modelled with and without including human-subsidized occurrences are likely to have different expressions when they are projected on the same geographical space [21], comparing the output of these models would allow estimating the extent to which man is contributing to the propagation of invasive plants, and this could be useful for developing control and management programs.

To test these hypotheses, we constructed high-resolution datasets for an invasive species widely propagated in Mexico, the Peruvian peppertree (*Schinus molle* L., Anacardiaceae). Unlike datasets commonly used to model climatic niches of invasive species, which mainly rely on data from literature and collections, our datasets were constructed from direct field observations. Further, in these datasets we discriminated between juvenile and adult peppertrees, as well as between the naturally established and man-planted individuals. These datasets were used to construct three climatic niche models. The first model was constructed with all occurrence records of peppertrees, irrespective of the life-cycle stage of individuals (juveniles or adults) and irrespective of whether these records belonged to naturally established individuals or man-planted trees. This model estimated the distribution of peppertrees under the influence of both, climate and human activities; for this reason, this model will be hereinafter referred to as *generalized climatic niche*. The second model was constructed with occurrence records of naturally established adult individuals only. This model was aimed to predict the distribution of mature peppertrees without including human subsides; hereinafter, this model will be referred to as *adult climatic niche*. Finally, the third model was constructed with occurrence records of naturally established juvenile individuals only, and this model corresponds to the *regeneration climatic niche* of our target species. The distributions of peppertrees predicted by these three models were compared under the following work hypotheses: (1) if predictions of the generalized climatic niche differ from those of the adult and regeneration climatic niches, then human intervention can be assumed to affect the distribution of peppertrees in Mexico; and (2) if predictions of the adult and the regeneration climatic niches differ, then adult and juvenile peppertrees can be assumed to have different climatic requirements for surviving.

## Materials and Methods

### Target species

The Peruvian peppertree is native to the Andean region of South America. This species was introduced into Africa, Asia, Europe, Oceania and North America, but Mexico is the country with the largest invasion history for this tree [20]. Peppertrees were introduced into the Valley of Mexico by the middle of the 16^th^ century as result of the commercial exchange between the former viceroyalties of Peru and New Spain [22]. Currently, they are still being used as ornamental species in most human settlements of Mexico, as well as hedgerows and windbreaks in rural areas [20]. However, it was recently indicated that peppertrees are colonizing abandoned agroecosystems, where naturally established adult trees can be found together with recruiting individuals [23].

### Species occurrence records and climatic variables

To obtain reliable information about the occurrence of Peruvian peppertrees in Mexico we conducted a series of field trips across this country between August 2012 and January 2014. Field trips included arid and semiarid environments, temperate and tropical forests, and riparian and coastal vegetation. The sampled area ranged from 32° N to 14° N of latitude and from 92° W to 116° W of longitude, covering about 80% of the continental surface of Mexico (1,567,398 km^2^). Sampling points were located on the side of highways and rural roads, always maintaining a minimum distance of 20 linear kilometres among them and a minimum distance of 10 km from any human settlement. At each sampling point, two well-trained observers looked for peppertrees within their visual ranges and geo-referenced those sites where the species was recorded (GPS Garmin Oregon 650, USA). Peppertrees recorded in these field sites were classified as naturally established or man-planted individuals. Naturally established peppertrees are easy to recognize because they grow isolated from conspecific individuals, usually associated with native plants, while man-planted peppertrees are always forming hedgerows or windbreaks [23]. Further, naturally established peppertrees were classified as adults (individuals over 2.0 m tall with flowers and/or fruits) or juveniles (young non-reproductive individuals up to 2.0 m tall). This discrimination of the data allowed us to discern between sites that have been naturally colonized by peppertrees and sites where their presence has been subsidized by human activities.

**Fig 1:**
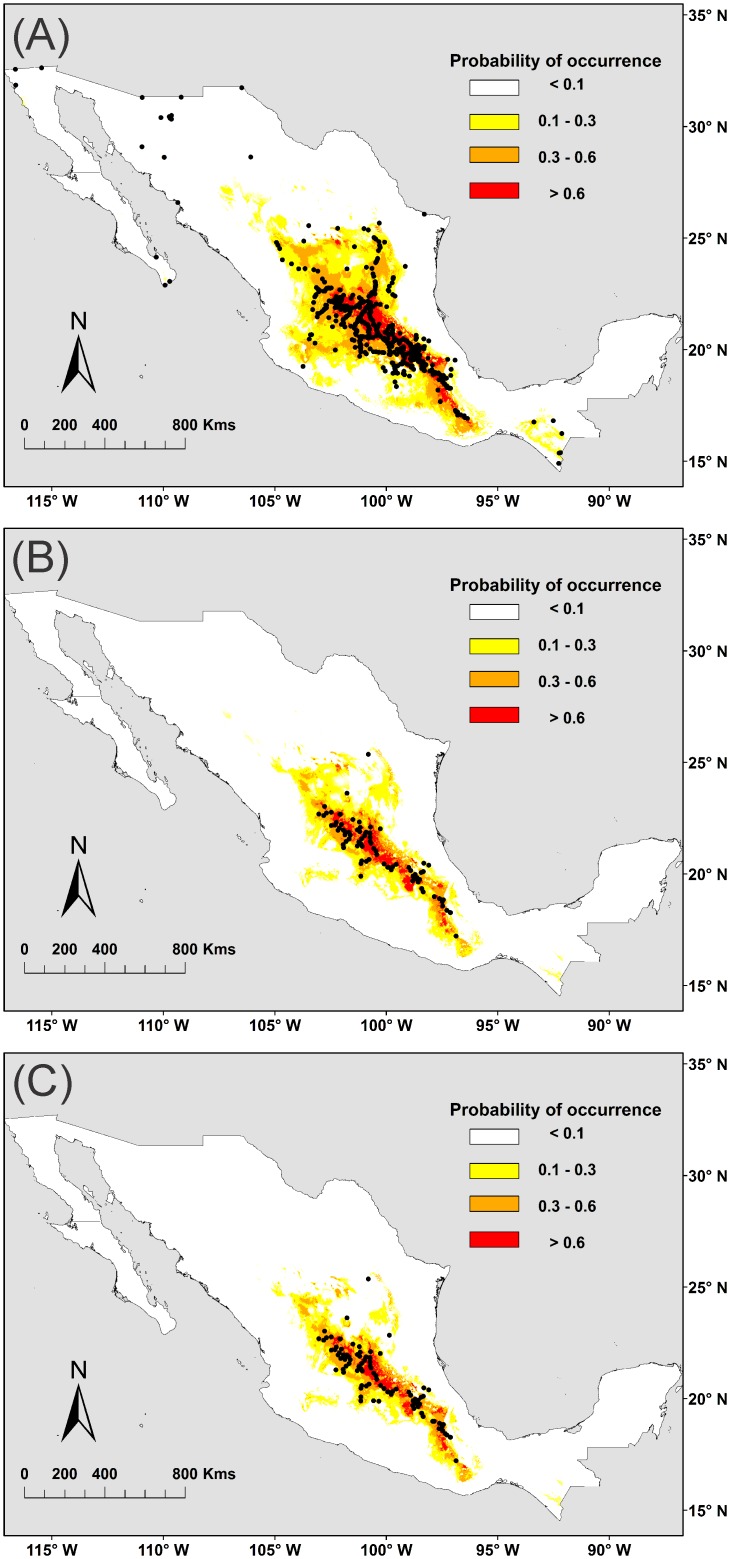
Distribution of occurrence data recorded for peppertrees in Mexico and the associated climatic niche models. The figure shows the generalized niche model calibrated with 307 occurrence points (A), the adult niche model calibrated with 98 occurrence points of naturally established adult peppertrees (B) and the regeneration niche model calibrated with 84 occurrence points of naturally established juvenile individuals (C). The base map of Mexico used for this figure was obtained from DIVA GIS (freely available for downloading and using at http://www.diva-gis.org/datadown).

Besides recording the presence of peppertrees in field sites, during the sampling trips we also recorded their presence in human settlements. In this case, we looked for peppertrees in yards, parks and streets [20]. Records from human settlements were added to those taken in field sites where man-planted peppertrees were recorded. We did it because, in both cases, the presence of peppertrees is subsidised by people. This sampling procedure resulted in 357 sites for man-panted peppertrees (Fig 1A), 97 sites for adult individuals naturally established in the field (Fig 1B), and 84 sites for juvenile individuals naturally established in the field (Fig 1C). It is important to note that naturally established juvenile peppertrees co-occurred with naturally established adult trees in 84% of the field sites (*i*.*e*. 82 of 97 field sites). Further, naturally established individuals (either juveniles or adults) co-occurred with man-planted peppertrees in 52% of the field sites (*i*.*e*. 51 of 97 field sites). These occurrence records are available in S1 Datasets (see Supporting Information).

Climatic variables of sites where the presence of peppertrees was recorded were obtained from the WorldClim layers (freely available at www.worldclim.org). These layers are interpolations of observed data, representative for the period 1950–2000, and provide information about 19 bioclimatic variables related to temperature and precipitation with a spatial resolution of 30 arc seconds (about 1-km^2^ per pixel) [24]. Although the samplings described above were conducted between 2012 and 2014, this climatic information is valuable for modelling the climatic niche of the Peruvian peppertree because most individuals that we recorded in the field are likely to be older than 10–20 years. The geographic background for this assessment covered the entire continental surface of Mexico and, given the strong disparity in elevation among sampling sites, altitude was included as additional geophysical variable. The resolution scale of this variable (altitude) was the same than that used for all the other bioclimatic variables (*i*.*e*. 30 arc seconds). Nevertheless, because these variables can be spatially auto-correlated [25], we firstly checked for cross-correlation between all possible pairs of variables with the Spearman correlation test, which was run in R 3.0 [26]. These correlation analyses allow to select variables for climatic niche models by minimizing redundancy within the full set of variables due to potential multicollinearity among them [27]. In these analyses, we look for relationships with correlation coefficients higher than 0.70 and when a given variable was related with several others, we only retained that variable that make more sense for explaining the distribution of plants [17,25,27,28].

### Climatic niche modelling

The generalized climatic niche of peppertrees was modelled including all sites where their presence was recorded, irrespective of the life-cycle stage of individuals (juveniles or adults) and their origin (naturally established or man-planted). For this model, however, it is important to note that some field sites contained more than one category in which peppertrees were classified (see above). For this reason, if naturally established individuals (either adults of juveniles) were found together with man-planted peppertrees, only the occurrence of man-planted individuals was retained for calibrating this model. This resulted in a total of 307 occurrence records that were used to calibrate this model. The adult climatic niche, on the other hand, was calibrated with the 97 occurrence records of naturally established adult peppertrees, while the regeneration climatic niche was modelled with the 84 occurrence records of naturally established juvenile individuals. In all cases, we also check for duplicate occurrences within each 30 arc seconds cell. For this, we used ArcGis 9.3 [29] to draw a 1-km circular plot around each occurrence point and, if two or more of these plots were overlapped, then one of the records were eliminated. This allowed us to control overestimations in the models.

All models were conducted with MaxEnt 3.3. This software is commonly used for modelling climatic niches of plant species, requiring a minimum of 50 occurrence data points to produce robust models [30]. MaxEnt computes the probability distribution of maximum entropy for the set of climatic variables associated to the occurrence records of the target species, but this procedure is constrained by the incomplete knowledge about the distribution of the species [30–32]. The resulting model is then a geographical projection of habitat suitability for the target species (*i*.*e*. probability for finding the species) where values close to 0 indicate sites that do not match with the niche requirements of the species, and values close to 1 indicate sites that fully match their niche requirements. Although other computer programs have also been used to model species climatic niches, several authors showed that MaxEnt usually performs better when presence-only data are available [31,32].

Each niche model was calibrated by randomly selecting 75% of the occurrence points from each dataset (training data), and the remaining 25% of the datasets was used to validate the models (test data). The accuracy of models for predicting the presence of the target species was tested with receiver operating characteristic curves (ROC), which were constructed by plotting the ratio of test data correctly classified by each climatic niche model (true positives) against the ratio of test points incorrectly classified by the model [33,34]. After that, the area under the ROC curves (AUC) was estimated to assess the accuracy of the models [29]. AUC values range between 0 and 1, where values below 0.5 indicate that the model resulted from random processes, while values close to 1 indicate that the model can accurately predict the distribution of the species [16,33]. These procedures were repeated 100 times, and the 100 models obtained from each dataset were averaged to calibrate the final climatic niche models [35,36]. Further, all models were regularized to avoid over-parameterization. In MaxEnt, this procedure relies in smoothing the models by modifying the value of a parameter (*β*) that controls the trade-off between complexity (number of variables) and predictive power of the models [35,36]. In our case, we always penalized over-parameterization by selecting the most conservative model with the best trade-off between complexity and predictive power (*i*.*e*. only models with *β* ≅ 1 were retained).

The final output of the generalized, adult and regeneration climatic niche models of peppertrees were visualized in DIVA-GIS 7.5 [37] and plotted in ArcGis 9.3 [30]. The lower 10% percentile of the models defined the threshold to discriminate between suitable and unsuitable habitats for the species–*i*.*e*. probability values below 0.1 indicate unsuitable climatic zones [36]. The accuracy of these final climatic niche models to predict the distribution of peppertrees was tested with the Boyce index (*B*). This index is computed as the ratio between the density of occurrence points falling within the thresholds of suitable habitat predicted by the model and the total number of sites where the species was recorded [11,38]. Values of *B* range between 0 and 1, where values close to 0 indicate that the model cannot predict the occurrence of the species and values close to 1 indicate elevated coincidence between the predictions of the model and the true distribution of the species [11,38]. To calculate this index, we over-imposed the occurrence data of peppertrees on the respective climatic niche model (generalized, adult and regeneration models) and computed the *B* values in R 3.0 [26]. Therefore, the Boyce index is complementary to AUC. However, while AUC only uses 25% of the data as validation points at each model run (100 runs for each model, in our case), the *B* index tests the robustness of the final models (i.e., the average of the 100 models) by including all occurrence points of the species [38].

### Comparison of niche models

Simple linear regression analyses were used to assess whether the generalized, adult and regeneration niche models coincided or differed in their predictions. For this, we generated 10,000 random pairs of geographic coordinates (latitude-longitude) in DIVA-GIS using the political bounds of Mexico as geographical background. These points were superimposed on each climatic niche model and we asked to MaxEnt the probability of finding peppertrees at each pair of coordinates.

Probability values obtained from the regeneration and adult niche models were regressed against the probability values obtained for the same pair of coordinates in the generalized niche model. Therefore, this method assesses the spatial correlation between predictions of the different models. Theoretically, if predictions of models concur, the intercepts of the empirical regression functions resulting from these analyses should be close to zero (0) and their slopes should approximate to one (1). Otherwise, if models predict different distributions for peppertrees, the parameters of these empirical regression functions should differ from their theoretical values. To perform these comparisons, we computed the 95% prediction intervals for the empirical regression functions and assessed whether the curve of the theoretical linear function (intercept = 0; slope = 1) was contained within these intervals. The same approach was later used to assess whether predictions of the regeneration niche model concurred with those of the adult niche model.

Although other methods have been proposed to compare niche models of plants [30], most of them have been designed to assess whether the environmental variables that constrain the distribution of species concur or differ between different geographic regions. In our case, since we were interested in comparing the output of different climatic niche models calibrated for the same geographical background, the regression analyses proposed above constitute a more intuitive and robust method to assess differences in their predictions. Indeed, including a large number of predicted values in these regression analyses (in our case we requested 10,000 random values from each model) allows increasing the strength of the resulting relationships. This allows to control the bandwidth of the 95% prediction intervals and reduces potential biases when the theoretical linear regression function is compared with the empirical regression function. All regression analyses described in this section were conducted in R 3.0 [26].

### Ethics statement

Because all field sites where we recorded the presence of peppertrees belongs to private owners or “ejidos” (a communal land tenure system of Mexico), we always asked for permission before performing our samplings. No specific permissions were required in human settlements because most these occurrence records were taken in public properties (parks and streets). Occurrence records in private yards were only taken when peppertrees were easily visible without entering into the properties. Finally, since we were only interested in assessing the presence of this invasive species, this study neither involves nor affects endangered or protected species.

## Results

### Climatic niche models

A total of seven environmental variables were retained for constructing the climatic niche models of peppertrees after checking for autocorrelation among them (Table 1). Although we applied regularization procedures to reduce parametrization in the three climatic niche models, all these variables were indicated as important predictors of the distribution of this invasive species in Mexico (*β* generalized niche model = 0.9998; *β* adult niche model = 0. 9999; *β* regeneration niche model = 0. 9999). The variable that better correlated with the distribution of peppertrees in Mexico was isothermality, which explained more than 30% of variance in the three niche models (Table 1). It was followed by annual precipitation and annual mean temperature, which explained more than 17% each, as compared to the other variables included in the models (Table 1). The remaining variables had explicative powers below 13% (Table 1), but all of them were retained in the models as important predictors of the distribution of peppertrees in Mexico.

**Table 1:** Environmental variables retained to model the climatic niches of the Peruvian peppertree in Mexico. The table shows the relative contribution of each variable to explain variance in each model (generalized, adult and regeneration niche models).

Variable	Generalized niche model	Adult niche model	Regeneration niche model
Annual Mean Temperature	17.6%	18.5%	19.4%
Mean Diurnal Range of Temperature	2.8%	3.1%	3.9%
Isothermality	40.1%	30.4%	31.4%
Annual Precipitation	21.4%	22.5%	22.7%
Precipitation Seasonality	4.5%	12.4%	12.3%
Precipitation-Driest Month	2.1%	1.2%	1.6%
Precipitation-Coldest Quarter of the Year	11.5%	11.9%	8.7%

The generalized niche model predicted the presence of peppertrees from 15° N to 27° N of latitude and from 97° W to 103° W of longitude (Fig 1A), covering up to 24.0% of the continental surface of Mexico (470445.4 km^2^). Occurrence probabilities above 0.6 were predicted in the central portion of this country and these values gradually decreased towards all cardinal directions (Fig 1A). The average AUC for this model was 0.9130 (±0.0040 SD) and its Boyce index was 0.9225.

The adult and the regeneration niche models predicted more restricted distributions for peppertrees than the generalized niche model. The adult niche model (Fig 1B) predicted the presence of this species from 15° N to 27° N of latitude and from 92° W to 107° W of longitude (11.9% of the continental surface of Mexico = 233262.5 km^2^), while the regeneration niche model (Fig 1C) ranged from 15° N to 26° N of latitude and from 92° W to 105° W of longitude (11.2% of the continental surface of Mexico = 219541.2 km^2^). In both cases, occurrence probabilities above 0.6 were concentrated in central Mexico (Fig 1B and 1C). The AUC for the adult niche model averaged 0.9730 (±0.0030 SD), while the regeneration niche model averaged 0.9760 (±0.0030 SD). The values of the Boyce index were 0.89906 and 0.8899 for adult and the regeneration niche model, respectively.

### Comparison of niche models

**Fig 2:**
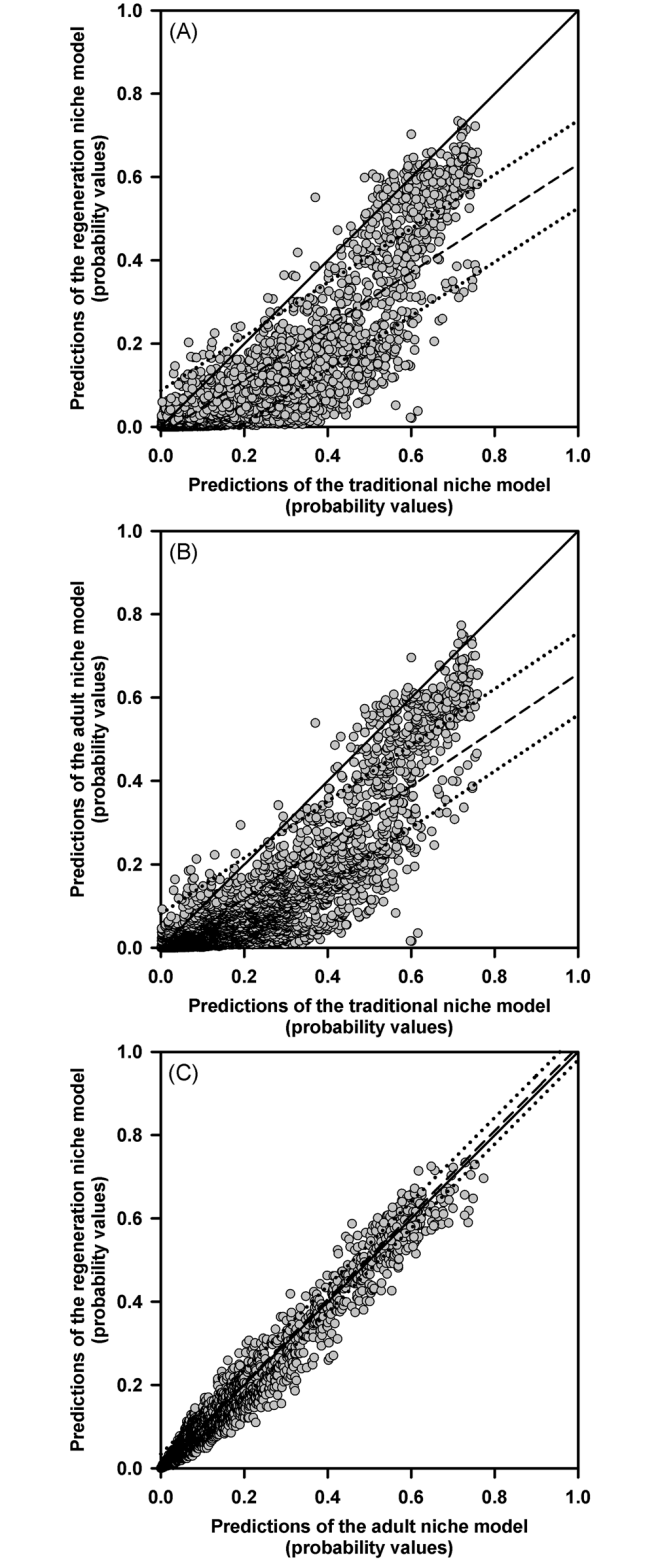
Relationships obtained from comparing predictions of the generalized niche model against predictions of the regeneration niche model (A) and predictions of the adult niche model (B). The figure also shows the relationship obtained by comparing the predictions of the adult and the regeneration niche models (C). Each panel shows the theoretical regression curve expected for perfect matching between predictions of models (solid line) and the empirical curves obtained from the regression analyses (dashed lines) with their 95% prediction intervals (dotted lines).

Occurrence probabilities predicted by the generalized niche model were positively related with those predicted by both, the regeneration niche model (*F*_(1,9998)_ = 45192.8437, *p* < 0.0001, *R*^2^ = 0.8188; Fig 2A) and the adult niche model (*F*_(1,9998)_ = 36831.8906, *p* < 0.0001, *R*^2^ = 0.7865; Fig 2B). The intercept of the empirical regression function obtained from comparing the generalized and regeneration niche models was -0.0178 and its slope was 0.6748. The comparison between the generalized and adult niche models resulted in an intercept of -0.0179 and a slope of 0.6486. In both cases, the 95% prediction intervals of the empirical regression functions did not contain the theoretical curve with intercept 0 and slope 1 (Fig 2A and 2B). Thus, the distribution of peppertrees predicted by the adult and regeneration niche models can be assumed to differ from that predicted by the generalized niche model.

A positive relationship was also found between predictions of the adult and the regeneration niche models (*F*_(1,9998)_ = 522236.3437, *p* < 0.0001, *R*^2^ = 0.9812; Fig 2C). The intercept of this regression function was 0.0022 and its slope was 1.0101. In this case, the 95% prediction intervals of the empirical regression function fully contained the theoretical curve with intercept 0 and slope 1 (Fig 2C). Thus, the adult and the regeneration niche models are assumed to concur in their predictions about the distribution of naturally established peppertrees in Mexico.

## Discussion

### Climatic niche models

Despite our efforts to regularize the climatic niches modelled for the Peruvian peppertree (*i*.*e*. by using *β* values ≅ 1 in MaxEnt), the seven environmental variables selected to calibrate them (see Table 1) were retained as important factors that predict its distribution in Mexico. This elevated number of bioclimatic variables retained in the models suggests that the spread of this invasive species results from complex interactions with the environment. Further, it is also important to consider that sampling sites used to record the presence of adult and juvenile individuals in the field were located on potential dispersal corridors for invasive species (e.g., roads), and this may also influence the output of these climatic niche models. Nevertheless, the elevated AUC values and Boyce indices obtained for the three climatic niche models suggest that all of them can robustly predict the distribution of peppertrees in Mexico. This concurs with the widely accepted proposal that bioclimatic variables and MaxEnt algorithms are useful to assess the distribution of plant species on large geographical scales [10,12,39]. However, despite the robustness of these models, the generalized climatic niche led to different predictions than those obtained from the adult and the regeneration niche models.

### Niche models and the spread of peppertrees

The lack of concurrence between predictions of the generalized climatic niche and those obtained from the adult and the regeneration niche models suggests that the current distribution of this invasive tree in Mexico would not be fully explained by climatic constraints. Indeed, the generalized niche model predicted a broader distribution range for peppertrees than the other two models. This suggests that this invasive species might occur in sites located farther beyond the climatic thresholds that naturally established individuals can tolerate. These results contradict the overall proposal that plant species should only occur in sites where environmental conditions allow the establishment of their early life-cycle stages [1–3,8]. Nevertheless, in our case, this situation may occur because humans are strongly influencing the spread of peppertrees in Mexico, where this species has been widely propagated throughout the country because of its rapid growth to obtain raw materials [20,40]. Further, after their introduction, peppertrees were quickly incorporated into the local culture because of their ethnobotanical uses in traditional medicine and religious ceremonies of native people [41–43]. Thus, after five centuries of invasion history, most Mexican people believe that the Peruvian peppertree is native to this country and continue subsidizing its establishment in human settlements and agroecosystems [20,23]. This strong link between peppertrees and the Mexican culture makes feasible to propose that the generalized climatic niche is overpredicting distribution range of this species because it included human-subsidized occurrence records. Thus, human activities could be artificially inflating the ecological niche of the species by promoting its occurrence outside the geographical range that peppertrees can naturally colonize according to their climatic niche requirements.

The adult and regeneration climatic niche models, on the other hand, concurred in predicting that the distribution of naturally established peppertrees (*i*.*e*. without including the influence of human subsides) is restricted to central Mexico. Indeed, when predictions of these two models were compared, the results indicated that the distribution of both adult and juvenile individuals would be regulated by similar climatic constrains. These results somewhat contradict the proposal that the niche requirements of plants change along their life cycles [1,3]. Nevertheless, it is important to note that adult and juvenile stages co-occurred in most field sites where the presence of the species was recorded. Thus, although different datasets were used to construct the adult and the regeneration niche models (naturally established adult and juvenile individuals, respectively), their predictions are likely to be spatially autocorrelated because of the elevated co-occurrence of adult and juvenile plants. However, rather than failing in estimating the climatic niche requirements of adult and juvenile peppertrees, this elevated concurrence in the predictions of these models may have important implications to understand how this biological invasion is naturally spreading in Mexico.

For tree species it was proposed that, once individuals reach maturity, they can induce environmental changes in colonized sites and make them unsuitable for the recruitment of conspecific individuals [3]. However, in our case, the concurring predictions of the adult and regeneration climatic niche models suggest that this would not be the case of peppertrees in central Mexico. This may be occurring because, even if the regeneration and adult climatic niches of peppertrees differ, perhaps the density of mature individuals in these sites is not large enough to locally reduce the availability of suitable microhabitats matching the climatic requirements of juvenile stages. Nevertheless, field experiments assessing seed germination and seedling establishment within monospecific peppertree stands are still being required to determine whether adult trees can interfere with the recruitment of their earlier life-cycle stages.

The concurring predictions of the adult and regeneration niche models also suggest that this biological invasion cannot expand by itself beyond central Mexico. In other words, if human intervention is not taken into account, these niche models indicate that the maximum distribution range that peppertrees can reach is constrained to this fraction of the country because climatic conditions outside this region would neither allow their survival nor their recruitment. Nevertheless, because man-planted peppertrees co-occurred with naturally established trees in several field sites within this region, it is feasible to propose that human-subsidized trees might be contributing to support the natural populations of the species in central Mexico. The elevated concurrence between predictions of the adult and regeneration niche models also suggests that naturally established peppertrees may have already attained the geographical equilibrium in Mexico–*i*.*e*. the species has colonized all sites that meet its climatic requirements [44–46]. This suggestion is supported by the lacking of naturally established individuals outside central Mexico, which in turn also allows proposing that man-planted individuals cannot develop viable populations beyond this region. However, although the adult and regeneration niche models seem to robustly predict the distribution of naturally established peppertrees, field experimentation would be required to test whether unfavourable climate effectively prevents the spread of this invasive species beyond the geographical bounds predicted by these models.

Aa final remark, it is important to highlight that this study indicates that climatic niche models constructed with data of naturally established plants can be useful to discern the specific role of climate on the spread of biological invasions. Such a modelling procedure would avoid the “noise” due to human intervention and concurs with the proposal of Sax *et al*. [21] about the *tolerance niche* of plant species. These authors proposed that the tolerance niche embrace those environmental factors that allow plants to live and grow only, while prevent the establishment of self-sustaining populations. The geographical protection of these niche requirements would then define the *tolerance distribution* of the species [21] and, by following this line of reasoning, we propose that human influence is an additional factor within the tolerance niche of peppertrees in Mexico.

## Conclusions

Our results suggest that modelling the regeneration climatic niche of invasive plants can constitute a useful tool for predicting their potential distributions. Nevertheless, developing these models requires high-resolution occurrence data, which must discriminate between naturally established individuals and human-induced presences. The application of this approach in areas invaded by aggressive plant species provides new opportunities for analysing whether their spread is mainly regulated by environmental conditions or by human activities. In turn, because the successful management of invasive plants mainly depends on our ability to predict their potential distributions, this approach also provides novel applications for niche-based distribution models. For example, if we are able to determine what areas are highly prone to be naturally invaded by alien plants, the economic efforts addressed to eradicate them could be focused in these sites. On the other hand, in those sites that are not likely to be naturally colonised by invasive plants, their spread could be controlled by applying educative programmes addressed to sensitize people about the risk that these species might represent for the environment.

## Supporting Information

S1 Datasets Occurrence records of Peruvian peppertrees in Mexico. (PDF)
