# Supplementary material for: Improved Predictions of the Geographic Distribution of Invasive Plants Using Climatic Niche Models
Source: PLoS One. 2016 May 19;11(5):e0156029. doi: 10.1371/journal.pone.0156029 (PMC4873032; doi:10.1371/journal.pone.0156029)
Supplement: S1 Datasets — (PDF) [file pone.0156029.s001.pdf]

S1 Datasets

Occurrence records of Peruvian peppertrees in Mexico (geographic coordinates). The datasets below include adult individuals naturally established in the field (Dataset 1), juvenile (non-reproductive) individuals naturally established in the field (Dataset 2), and human-subsidized occurrences in human settlements and field sites where man-planted peppertrees were found (Dataset 3).

Dataset 1. Occurrence records of naturally established adult peppertrees (individuals over 2.0 m tall with flowers and/or fruits) taken in field sites from Mexico.

**Occurrence record**	**Latitude**	**Longitude**
Occurrence 001	21.9796	-101.2886
Occurrence 002	21.8731	-101.6726
Occurrence 003	21.8001	-102.0365
Occurrence 004	21.9101	-101.8622
Occurrence 005	21.9652	-101.9186
Occurrence 006	21.7596	-101.8473
Occurrence 007	21.9024	-101.1897
Occurrence 008	22.3319	-101.2017
Occurrence 009	22.4444	-101.4911
Occurrence 010	23.6217	-101.7567
Occurrence 011	22.6719	-102.0580
Occurrence 012	22.7678	-102.4644
Occurrence 013	23.0298	-102.7611
Occurrence 014	21.2894	-102.2542
Occurrence 015	20.5955	-100.2505
Occurrence 016	20.3042	-99.9343
Occurrence 017	21.8485	-100.7447
Occurrence 018	21.4672	-100.7172
Occurrence 019	21.0334	-100.4614
Occurrence 020	18.9870	-97.9135
Occurrence 021	18.6574	-97.6327
Occurrence 022	18.2718	-97.1137
Occurrence 023	25.3641	-100.7992
Occurrence 024	22.8455	-99.8554
Occurrence 025	22.1024	-100.6997
Occurrence 026	22.0253	-100.2641
Occurrence 027	21.8736	-100.8960
Occurrence 028	21.7229	-100.9662
Occurrence 029	21.5778	-101.0984
Occurrence 030	21.4384	-101.2216
Occurrence 031	21.2552	-101.2931
Occurrence 032	21.3140	-101.9278

Dataset 1 (continued).

Occurrence record	Latitude	Longitude
Occurrence 033	21.4587	-101.7972
Occurrence 034	21.5758	-101.6630
Occurrence 035	21.7473	-101.6076
Occurrence 036	22.0846	-101.1644
Occurrence 037	22.6760	-102.0805
Occurrence 038	22.7336	-102.6780
Occurrence 039	22.6208	-102.8000
Occurrence 040	22.6854	-103.0207
Occurrence 041	22.1738	-102.4571
Occurrence 042	22.1929	-102.2947
Occurrence 043	22.3138	-102.2363
Occurrence 044	22.2232	-102.0014
Occurrence 045	22.0554	-102.0990
Occurrence 046	21.9608	-102.1591
Occurrence 047	18.3684	-97.2801
Occurrence 048	21.8523	-100.9138
Occurrence 049	21.3511	-101.1364
Occurrence 050	21.2444	-101.0720
Occurrence 051	21.1797	-101.9089
Occurrence 052	21.9558	-101.7623
Occurrence 053	20.6540	-100.7661
Occurrence 054	20.5697	-100.8889
Occurrence 055	20.5895	-101.0978
Occurrence 056	20.4689	-101.1314
Occurrence 057	20.0821	-101.1371
Occurrence 058	19.9035	-101.1360
Occurrence 059	19.9063	-100.5691
Occurrence 060	19.8868	-100.3267
Occurrence 061	19.6927	-98.8143
Occurrence 062	19.8266	-98.7062
Occurrence 063	19.9324	-98.5648
Occurrence 064	20.0673	-98.4310
Occurrence 065	21.1476	-100.5641
Occurrence 066	20.8716	-100.4217
Occurrence 067	20.1891	-99.7728
Occurrence 068	21.8318	-100.7305
Occurrence 069	21.6400	-100.7211
Occurrence 070	21.3784	-100.6797
Occurrence 071	20.4577	-100.0923
Occurrence 072	18.9876	-97.8433
Occurrence 073	18.8902	-97.6008
Occurrence 074	18.8454	-97.4375
Occurrence 075	17.2210	-96.8676
Occurrence 076	18.2739	-97.1287
Occurrence 077	18.4257	-97.3485

Dataset 1 (continued).

Occurrence record	Latitude	Longitude
Occurrence 078	18.5654	-97.4638
Occurrence 079	18.7657	-97.4836
Occurrence 080	18.8965	-97.6106
Occurrence 081	19.2023	-98.3408
Occurrence 082	19.3520	-98.4056
Occurrence 083	19.6393	-98.5633
Occurrence 084	19.7934	-98.6562
Occurrence 085	19.9083	-98.8022
Occurrence 086	20.1096	-98.3323
Occurrence 087	19.7610	-98.5007
Occurrence 088	19.8329	-98.6112
Occurrence 089	19.9803	-98.7020
Occurrence 090	20.1568	-98.9018
Occurrence 091	20.2963	-98.9835
Occurrence 092	20.3950	-98.0898
Occurrence 093	20.4846	-98.3143
Occurrence 094	20.4326	-99.5501
Occurrence 095	20.3208	-99.7108
Occurrence 096	20.3040	-99.8929
Occurrence 097	19.5578	-98.7521

Dataset 2. Occurrence records of naturally established juvenile peppertrees (young non-reproductive individuals up to 2.0 m tall) taken in field sites from Mexico.

Occurrence record	Latitude	Longitude
Occurrence 001	21.9796	-101.2886
Occurrence 002	21.8731	-101.6726
Occurrence 003	21.9101	-101.8622
Occurrence 004	21.9652	-101.9186
Occurrence 005	21.7596	-101.8473
Occurrence 006	21.9024	-101.1897
Occurrence 007	22.3319	-101.2017
Occurrence 008	22.4444	-101.4911
Occurrence 009	23.6217	-101.7567
Occurrence 010	22.6719	-102.0580
Occurrence 011	22.7678	-102.4644
Occurrence 012	23.0298	-102.7611
Occurrence 013	20.3042	-99.9343
Occurrence 014	21.8485	-100.7447
Occurrence 015	21.4672	-100.7172
Occurrence 016	21.0334	-100.4614
Occurrence 017	18.2718	-97.1137
Occurrence 018	25.3641	-100.7992
Occurrence 019	22.1024	-100.6997

Dataset 2 (continued).

Occurrence record	Latitude	Longitude
Occurrence 020	22.0253	-100.2641
Occurrence 021	21.8736	-100.8960
Occurrence 022	21.7229	-100.9662
Occurrence 023	21.5778	-101.0984
Occurrence 024	21.4384	-101.2216
Occurrence 025	21.2552	-101.2931
Occurrence 026	21.3140	-101.9278
Occurrence 027	21.4587	-101.7972
Occurrence 028	21.5758	-101.6630
Occurrence 029	21.7473	-101.6076
Occurrence 030	22.0846	-101.1644
Occurrence 031	22.6760	-102.0805
Occurrence 032	22.7336	-102.6780
Occurrence 033	22.6208	-102.8000
Occurrence 034	22.6854	-103.0207
Occurrence 035	22.1738	-102.4571
Occurrence 036	22.1929	-102.2947
Occurrence 037	22.3138	-102.2363
Occurrence 038	22.2232	-102.0014
Occurrence 039	22.0554	-102.0990
Occurrence 040	21.9608	-102.1591
Occurrence 041	18.3684	-97.2801
Occurrence 042	21.8523	-100.9138
Occurrence 043	21.3511	-101.1364
Occurrence 044	21.2444	-101.0720
Occurrence 045	21.1797	-101.9089
Occurrence 046	20.6540	-100.7661
Occurrence 047	20.5697	-100.8889
Occurrence 048	20.5895	-101.0978
Occurrence 049	20.4689	-101.1314
Occurrence 050	19.9035	-101.1360
Occurrence 051	19.6927	-98.8143
Occurrence 052	19.8266	-98.7062
Occurrence 053	20.0673	-98.4310
Occurrence 054	21.1476	-100.5641
Occurrence 055	20.8716	-100.4217
Occurrence 056	20.1891	-99.7728
Occurrence 057	21.8318	-100.7305
Occurrence 058	21.6400	-100.7211
Occurrence 059	21.3784	-100.6797
Occurrence 060	20.4577	-100.0923
Occurrence 061	18.9876	-97.8433
Occurrence 062	18.8902	-97.6008
Occurrence 063	18.8454	-97.4375

Dataset 2 (continued).

Occurrence record	Latitude	Longitude
Occurrence 064	17.2210	-96.8676
Occurrence 065	18.2739	-97.1287
Occurrence 066	18.5654	-97.4638
Occurrence 067	18.7657	-97.4836
Occurrence 068	18.8965	-97.6106
Occurrence 069	19.2023	-98.3408
Occurrence 070	19.3520	-98.4056
Occurrence 071	19.6393	-98.5633
Occurrence 072	19.7934	-98.6562
Occurrence 073	19.9083	-98.8022
Occurrence 074	20.1096	-98.3323
Occurrence 075	19.7610	-98.5007
Occurrence 076	19.8329	-98.6112
Occurrence 077	19.9803	-98.7020
Occurrence 078	20.1568	-98.9018
Occurrence 079	20.2963	-98.9835
Occurrence 080	20.3950	-98.0898
Occurrence 081	20.4846	-98.3143
Occurrence 082	20.4326	-99.5501
Occurrence 083	20.3208	-99.7108
Occurrence 084	20.3040	-99.8929

Dataset 3. Occurrence records of peppertrees taken in human settlements and field sites where man-planted individuals were found.

Occurrence record	Latitude	Longitude
Occurrence 001	14.9058	-92.2632
Occurrence 002	15.3688	-92.2448
Occurrence 003	16.2439	-92.1242
Occurrence 004	16.7574	-93.3737
Occurrence 005	16.8190	-92.5061
Occurrence 006	16.9227	-96.3625
Occurrence 007	16.9543	-96.4739
Occurrence 008	17.0305	-96.5200
Occurrence 009	17.0667	-96.7895
Occurrence 010	17.0812	-96.6606
Occurrence 011	17.2856	-96.9024
Occurrence 012	17.6728	-97.5723
Occurrence 013	18.1898	-97.6791
Occurrence 014	18.2603	-97.1510
Occurrence 018	18.3684	-97.2801
Occurrence 019	18.3763	-97.2878
Occurrence 020	18.4034	-97.3297
Occurrence 021	18.4257	-97.3485

Dataset 3 (continued).

Occurrence record	Latitude	Longitude
Occurrence 022	18.4444	-97.3690
Occurrence 023	18.4648	-97.3865
Occurrence 024	18.5563	-99.6053
Occurrence 025	18.5654	-97.4638
Occurrence 015	18.2938	-97.2708
Occurrence 016	18.3251	-97.2406
Occurrence 017	18.3455	-99.5397
Occurrence 026	18.6574	-97.6327
Occurrence 027	18.7157	-97.3078
Occurrence 028	18.7657	-97.4836
Occurrence 029	18.8141	-98.9547
Occurrence 030	18.8351	-97.5463
Occurrence 031	18.8784	-99.1784
Occurrence 032	18.8902	-97.6008
Occurrence 033	18.9134	-97.8238
Occurrence 034	18.9231	-99.2265
Occurrence 035	18.9348	-99.2322
Occurrence 036	18.9439	-99.4943
Occurrence 037	18.9560	-97.6582
Occurrence 038	18.9611	-99.5903
Occurrence 039	18.9788	-97.7851
Occurrence 040	19.0344	-99.2052
Occurrence 041	19.0402	-98.0466
Occurrence 042	19.0436	-100.0420
Occurrence 043	19.0527	-97.9925
Occurrence 044	19.0626	-98.2774
Occurrence 045	19.0829	-98.3195
Occurrence 046	19.1032	-99.5903
Occurrence 047	19.1065	-97.9537
Occurrence 048	19.1176	-98.7680
Occurrence 049	19.1216	-97.0846
Occurrence 050	19.1576	-98.4085
Occurrence 051	19.1617	-99.6178
Occurrence 052	19.1959	-100.1327
Occurrence 053	19.2023	-98.3408
Occurrence 054	19.2329	-98.3144
Occurrence 055	19.2512	-103.7303
Occurrence 056	19.2563	-99.6048
Occurrence 057	19.2572	-98.4059
Occurrence 058	19.2667	-98.9000
Occurrence 059	19.2724	-98.8358
Occurrence 060	19.2811	-99.4806
Occurrence 061	19.2814	-98.9030
Occurrence 062	19.3001	-98.2401

Dataset 3 (continued).

Occurrence record	Latitude	Longitude
Occurrence 063	19.3130	-97.9240
Occurrence 064	19.3297	-98.2179
Occurrence 065	19.3491	-99.0542
Occurrence 066	19.3520	-98.4056
Occurrence 067	19.3616	-99.3502
Occurrence 068	19.4004	-99.6959
Occurrence 069	19.4108	-99.7296
Occurrence 070	19.4203	-99.1819
Occurrence 071	19.4697	-98.5566
Occurrence 072	19.4706	-98.3471
Occurrence 073	19.4840	-99.2424
Occurrence 074	19.5126	-98.8800
Occurrence 075	19.5305	-96.9091
Occurrence 076	19.5406	-98.5565
Occurrence 077	19.5514	-98.8104
Occurrence 078	19.5629	-97.2416
Occurrence 079	19.5781	-98.6769
Occurrence 080	19.5904	-98.6189
Occurrence 081	19.6206	-98.1239
Occurrence 082	19.6393	-98.5633
Occurrence 083	19.6450	-99.1694
Occurrence 084	19.6850	-98.8660
Occurrence 085	19.6985	-98.7537
Occurrence 086	19.7015	-101.1868
Occurrence 087	19.7055	-98.8376
Occurrence 088	19.7171	-99.2230
Occurrence 089	19.7412	-98.4880
Occurrence 090	19.7436	-99.1814
Occurrence 091	19.7610	-98.5007
Occurrence 092	19.7759	-98.7123
Occurrence 093	19.7761	-99.2092
Occurrence 094	19.7775	-98.5454
Occurrence 095	19.7839	-99.8150
Occurrence 096	19.7863	-98.5523
Occurrence 097	19.7944	-99.8673
Occurrence 098	19.8096	-100.8912
Occurrence 099	19.8266	-98.7062
Occurrence 100	19.8329	-98.6112
Occurrence 101	19.8331	-99.2654
Occurrence 102	19.8344	-98.9835
Occurrence 103	19.8478	-98.6691
Occurrence 104	19.8532	-98.7459
Occurrence 105	19.8655	-100.1051
Occurrence 106	19.8760	-100.1771

Dataset 3 (continued).

Occurrence record	Latitude	Longitude
Occurrence 107	19.8817	-100.9460
Occurrence 108	19.8892	-98.6553
Occurrence 109	19.8924	-99.3465
Occurrence 110	19.9001	-99.3417
Occurrence 111	19.9035	-101.1360
Occurrence 112	19.9063	-100.5691
Occurrence 113	19.9083	-98.8022
Occurrence 114	19.9424	-99.3892
Occurrence 115	19.9545	-99.8436
Occurrence 116	19.9684	-98.5186
Occurrence 117	19.9692	-101.1397
Occurrence 118	19.9803	-98.7020
Occurrence 119	19.9864	-99.3199
Occurrence 120	19.9882	-102.2854
Occurrence 121	19.9889	-98.7084
Occurrence 122	20.0059	-99.2297
Occurrence 123	20.0338	-100.7212
Occurrence 124	20.0573	-98.4935
Occurrence 125	20.0584	-99.2206
Occurrence 126	20.0821	-101.1371
Occurrence 127	20.0835	-98.3676
Occurrence 128	20.0905	-101.1580
Occurrence 129	20.1096	-98.3323
Occurrence 130	20.1297	-99.2289
Occurrence 131	20.1355	-98.8856
Occurrence 132	20.1412	-101.1809
Occurrence 133	20.1488	-99.7432
Occurrence 134	20.1495	-98.2858
Occurrence 135	20.1694	-98.6488
Occurrence 136	20.1790	-99.7630
Occurrence 137	20.1891	-99.7728
Occurrence 138	20.2069	-98.8932
Occurrence 139	20.2122	-101.1330
Occurrence 140	20.2230	-99.8157
Occurrence 141	20.2684	-98.9279
Occurrence 142	20.2775	-99.9308
Occurrence 143	20.2849	-99.4124
Occurrence 144	20.2950	-103.1899
Occurrence 145	20.2963	-98.9835
Occurrence 146	20.3042	-99.9343
Occurrence 147	20.3100	-101.1788
Occurrence 148	20.3290	-99.9366
Occurrence 149	20.3882	-99.0701
Occurrence 150	20.3943	-101.1926

Dataset 3 (continued).

Occurrence record	Latitude	Longitude
Occurrence 151	20.3950	-98.0898
Occurrence 152	20.4026	-100.0279
Occurrence 153	20.4095	-99.3484
Occurrence 154	20.4143	-101.4339
Occurrence 155	20.4375	-99.5319
Occurrence 156	20.4423	-99.1722
Occurrence 157	20.4512	-101.5267
Occurrence 158	20.4577	-100.0923
Occurrence 159	20.4663	-98.6726
Occurrence 160	20.4689	-101.1314
Occurrence 161	20.4723	-103.4475
Occurrence 162	20.4723	-100.1051
Occurrence 163	20.4749	-99.2119
Occurrence 164	20.4846	-98.3143
Occurrence 165	20.4979	-99.9293
Occurrence 166	20.5121	-100.0856
Occurrence 167	20.5223	-100.8122
Occurrence 168	20.5329	-99.6329
Occurrence 169	20.5339	-101.1253
Occurrence 170	20.5512	-100.5837
Occurrence 171	20.5697	-100.8889
Occurrence 172	20.5924	-101.1262
Occurrence 173	20.5955	-100.2505
Occurrence 174	20.6314	-100.4285
Occurrence 175	20.6460	-98.6540
Occurrence 176	20.6540	-100.7661
Occurrence 177	20.6668	-103.3918
Occurrence 178	20.6721	-99.8947
Occurrence 179	20.6741	-103.3445
Occurrence 180	20.6791	-101.3546
Occurrence 181	20.6791	-100.7647
Occurrence 182	20.6870	-99.7345
Occurrence 183	20.7006	-100.7715
Occurrence 184	20.7035	-99.7205
Occurrence 185	20.7064	-100.3456
Occurrence 186	20.7252	-101.3737
Occurrence 187	20.7324	-99.9460
Occurrence 188	20.7504	-100.7703
Occurrence 189	20.7527	-99.7183
Occurrence 190	20.7762	-100.4099
Occurrence 191	20.7835	-99.7211
Occurrence 192	20.8000	-101.3335
Occurrence 193	20.8022	-100.4104
Occurrence 194	20.8162	-101.3138

Dataset 3 (continued).

**Occurrence record**	**Latitude**	**Longitude**
Occurrence 195	20.8231	-100.4076
Occurrence 196	20.8316	-99.7197
Occurrence 197	20.8511	-100.7788
Occurrence 198	20.8545	-101.3074
Occurrence 199	20.8829	-99.7066
Occurrence 200	20.8855	-100.4165
Occurrence 201	20.9192	-100.7448
Occurrence 202	20.9367	-100.4173
Occurrence 203	20.9971	-100.3873
Occurrence 204	21.0054	-100.4377
Occurrence 205	21.0214	-101.8537
Occurrence 206	21.0257	-99.7444
Occurrence 207	21.0302	-102.4044
Occurrence 208	21.0416	-99.8655
Occurrence 209	21.0492	-100.7974
Occurrence 210	21.0494	-99.7775
Occurrence 211	21.0540	-101.5814
Occurrence 212	21.0586	-99.8025
Occurrence 213	21.0675	-100.8029
Occurrence 214	21.0884	-101.3944
Occurrence 215	21.1009	-99.7113
Occurrence 216	21.1247	-100.8346
Occurrence 217	21.1250	-101.3842
Occurrence 218	21.1254	-100.5713
Occurrence 219	21.1299	-102.0019
Occurrence 220	21.1436	-100.3190
Occurrence 221	21.1521	-101.3770
Occurrence 222	21.1774	-100.9434
Occurrence 223	21.1797	-101.9089
Occurrence 224	21.2324	-101.0272
Occurrence 225	21.2894	-102.2542
Occurrence 226	21.3060	-100.5961
Occurrence 227	21.3328	-101.1239
Occurrence 228	21.3784	-100.6797
Occurrence 229	21.4285	-101.1843
Occurrence 230	21.4488	-101.8050
Occurrence 231	21.4672	-100.7172
Occurrence 232	21.4673	-103.0887
Occurrence 233	21.4751	-101.7878
Occurrence 234	21.4818	-100.7224
Occurrence 235	21.5017	-101.7514
Occurrence 236	21.5258	-102.2404
Occurrence 237	21.5778	-101.0984
Occurrence 238	21.5909	-101.0891
Occurrence 239	21.6066	-100.7616

Dataset 3 (continued).

**Occurrence record**	**Latitude**	**Longitude**
Occurrence 240	21.6125	-101.0780
Occurrence 241	21.6273	-103.0660
Occurrence 242	21.6329	-102.9843
Occurrence 243	21.6643	-100.7132
Occurrence 244	21.6974	-101.6143
Occurrence 245	21.7070	-102.8692
Occurrence 246	21.7137	-100.9797
Occurrence 247	21.7473	-101.6076
Occurrence 248	21.7596	-101.8473
Occurrence 249	21.7670	-100.6986
Occurrence 250	21.7749	-102.8038
Occurrence 251	21.7975	-103.2989
Occurrence 252	21.8170	-100.7303
Occurrence 253	21.8207	-102.7693
Occurrence 254	21.8318	-100.7305
Occurrence 255	21.8485	-100.7447
Occurrence 256	21.8654	-101.5936
Occurrence 257	21.8717	-102.7030
Occurrence 258	21.8736	-100.8960
Occurrence 259	21.8759	-100.7702
Occurrence 260	21.8817	-102.2909
Occurrence 261	21.8900	-101.6501
Occurrence 262	21.8914	-102.2158
Occurrence 263	21.9024	-101.1897
Occurrence 264	21.9035	-100.8711
Occurrence 265	21.9101	-101.8622
Occurrence 266	21.9204	-102.2049
Occurrence 267	21.9226	-99.9815
Occurrence 268	21.9272	-101.8576
Occurrence 269	21.9382	-100.5831
Occurrence 270	21.9467	-101.8592
Occurrence 271	21.9504	-102.1529
Occurrence 272	21.9647	-101.9522
Occurrence 273	21.9764	-100.8062
Occurrence 274	21.9781	-102.6772
Occurrence 275	22.0115	-101.2428
Occurrence 276	22.0388	-100.7330
Occurrence 277	22.0431	-102.1116
Occurrence 278	22.0578	-101.1817
Occurrence 279	22.0970	-100.7087
Occurrence 280	22.1006	-101.0914
Occurrence 281	22.1166	-102.0648
Occurrence 282	22.1568	-100.9862
Occurrence 283	22.1581	-102.4793
Occurrence 284	22.2232	-102.0014

Dataset 3 (continued).

Occurrence record	Latitude	Longitude
Occurrence 285	22.2657	-101.1126
Occurrence 286	22.2821	-101.9887
Occurrence 287	22.3138	-102.2363
Occurrence 288	22.3289	-101.1972
Occurrence 289	22.3481	-102.0691
Occurrence 290	22.3701	-103.2040
Occurrence 291	22.3869	-100.7784
Occurrence 292	22.4052	-99.6005
Occurrence 293	22.4179	-103.1712
Occurrence 294	22.4419	-101.9104
Occurrence 295	22.4483	-100.6794
Occurrence 296	22.4687	-99.7074
Occurrence 297	22.4996	-100.5000
Occurrence 298	22.5604	-100.6465
Occurrence 299	22.5922	-103.0251
Occurrence 300	22.6208	-102.8000
Occurrence 301	22.6313	-100.5241
Occurrence 302	22.6661	-99.9074
Occurrence 303	22.6844	-100.5058
Occurrence 304	22.6939	-103.0304
Occurrence 305	22.7336	-102.6780
Occurrence 306	22.7678	-102.4644
Occurrence 307	22.7889	-103.1313
Occurrence 308	22.8136	-100.4869
Occurrence 309	22.8455	-99.8554
Occurrence 310	22.8922	-109.9179
Occurrence 311	22.9160	-100.4692
Occurrence 312	22.9963	-99.7300
Occurrence 313	23.0298	-102.7611
Occurrence 314	23.0459	-100.4910
Occurrence 315	23.0612	-109.7061
Occurrence 316	23.1729	-102.8627
Occurrence 317	23.2372	-99.6770
Occurrence 318	23.2874	-100.5588
Occurrence 319	23.5227	-103.0656
Occurrence 320	23.5818	-103.2521
Occurrence 321	23.6217	-101.7567
Occurrence 322	23.6264	-103.9213
Occurrence 323	23.6903	-100.8861
Occurrence 324	23.7361	-99.1418
Occurrence 325	23.8483	-104.2442
Occurrence 326	23.9016	-100.4744
Occurrence 327	24.0123	-100.4126
Occurrence 328	24.0274	-104.6530
Occurrence 329	24.0923	-100.3818

Dataset 3 (continued).

Occurrence record	Latitude	Longitude
Occurrence 330	24.1445	-110.3105
Occurrence 331	24.4408	-100.3476
Occurrence 332	24.5288	-104.7745
Occurrence 333	24.5779	-100.2678
Occurrence 334	24.6144	-101.4191
Occurrence 335	24.6738	-100.2990
Occurrence 336	24.8075	-104.9163
Occurrence 337	24.8194	-100.0698
Occurrence 338	24.8668	-103.6967
Occurrence 339	25.0269	-100.5509
Occurrence 340	25.3641	-100.7992
Occurrence 341	25.4421	-102.1756
Occurrence 342	25.5582	-103.4722
Occurrence 343	25.6738	-100.3090
Occurrence 344	26.0703	-98.2911
Occurrence 345	26.5912	-109.3417
Occurrence 346	28.6237	-109.9545
Occurrence 347	28.6339	-106.0741
Occurrence 348	29.0920	-110.9502
Occurrence 349	30.3282	-109.6297
Occurrence 350	30.4000	-110.1000
Occurrence 351	30.4444	-109.7280
Occurrence 352	31.3007	-110.9400
Occurrence 353	31.3139	-109.1900
Occurrence 354	31.7371	-106.4747
Occurrence 355	31.8563	-116.6098
Occurrence 356	32.5678	-116.6283
Occurrence 357	32.6263	-115.4530
